# Risk Factors of Idiopathic Congenital Clubfoot in the South of Vietnam: A Hospital-Based Study

**DOI:** 10.7759/cureus.79049

**Published:** 2025-02-15

**Authors:** Nam Q Vo, Giam M Trinh

**Affiliations:** 1 Pediatric Orthopaedics, Hospital for Traumatology and Orthopaedics, Ho Chi Minh City, VNM; 2 Traumatology and Orthopaedics, Pham Ngoc Thach University of Medicine, Ho Chi Minh City, VNM

**Keywords:** breech presentation, congenital clubfoot, epidemiological characteristics, idiopathic congenital clubfoot, risk factor

## Abstract

Purpose: To date, there are no comprehensive epidemiological studies exploring congenital clubfoot (CCF) within the Vietnamese population. This study aimed to identify potential risk factors associated with idiopathic CCF in infants and their mothers in southern Vietnam.

Methods: A case-control study was conducted to compare the environmental and socio-demographic characteristics of mothers and infants between two groups: infants diagnosed with idiopathic CCF (cases) and those without any congenital deformity (controls). The study included 233 cases and 232 controls. All infants were born in 2002, and their mothers resided in the southern and highland provinces of Vietnam. Associations between maternal and infant characteristics and the occurrence of CCF were assessed using odds ratios and Fisher's exact test.

Results: The prevalence of clubfoot was significantly higher in male infants compared to females (OR = 1.75; 95% CI: 1.17-2.61). The breech presentation was strongly associated with an increased risk of clubfoot (p = 0.001). Mothers living in the western, eastern, and highland regions of Vietnam had significantly higher odds of having infants with clubfoot compared to those residing in Ho Chi Minh City, with odds ratios of 2.22 (95% CI: 1.39-3.55), 1.97 (95% CI: 1.21-3.23), and 3.86 (95% CI: 1.74-7.79), respectively.

Conclusions: This study identified significant associations between CCF and infant sex, breech presentation, and maternal geographical location. Further, population-based studies are needed to explore these risk factors on a broader scale.

## Introduction

Congenital clubfoot (CCF) is a complex deformity involving three-dimensional abnormalities of the ankle and foot which include equinus and varus deformities of the hindfoot, adductus and cavus deformities of the midfoot, and plantar concavity [1], and understanding the risk factors is crucial for early diagnosis and intervention. CCF may be associated with neuromuscular disorders or systemic syndromes, such as myelomeningocele or congenital arthrogryposis; however, most cases are idiopathic [1].

There are notable differences in the incidence of CCF among racial and ethnic groups, with rates of 6.8/1000 in Polynesian populations, 1.12/1000 in Caucasians, 0.76/1000 in individuals of Hispanic descent, 0.5/1000 in Japanese populations, and 0.39/1000 in Chinese populations [2]. Although nationwide statistics on CCF incidence in Vietnam are unavailable, some reports suggest an estimated rate of 1/1000 [3].

Numerous basic, clinical, and epidemiological studies have sought to elucidate the etiology and pathogenesis of CCF to prevent this common congenital deformity. However, etiology and pathogenesis remain unclear. Large-scale epidemiological studies have identified certain risk factors associated with CCF, notably male sex [4-9] and maternal smoking during pregnancy [5,6,10-13]. To date, there are no comprehensive epidemiological studies exploring CCF among Southeast Asian populations in general, or Vietnamese populations. In 2012, Nguyen et al. [14] conducted a case-control study in Vietnam to investigate the risk factors for idiopathic CCF. The study included 99 cases of idiopathic CCF and 97 controls consisting of children aged 0-18 years without congenital anomalies. The findings demonstrated a significantly increased risk of CCF in children born to mothers under 25 years of age (p = 0.026) and in Breech deliveries (p = 0.033). However, the study was hospital-based, with a relatively small and heterogeneous sample in terms of birth year and maternal residence.

Furthermore, the effects of Agent Orange exposure on the offspring of Vietnam War veterans have been unofficially documented, encompassing a range of congenital anomalies affecting internal organs, the central nervous system, and the musculoskeletal system, including CCF. However, these CCFs are pathologically associated with cerebral palsy, myelomeningocele, and other conditions.

This analytical epidemiological study aims to identify maternal and child characteristics associated with idiopathic CCF in southern Vietnam. It also serves as a feasible foundation for larger-scale epidemiological studies in Vietnam.

## Materials and methods

Subjects

Between 2004 and 2012, data were collected on 346 children with congenital clubfoot (CCF) born in 2003 or later at the Hospital for Traumatology and Orthopaedics by a research team comprising physicians specializing in clubfoot treatment. Cases excluded from the study included pathological congenital clubfoot associated with conditions such as congenital arthrogryposis, myelomeningocele, or suspected pathological clubfoot (often accompanied by other anomalies), twins, mothers residing outside the study region, cases where the primary caregiver was not the biological mother (due to the potential inaccuracy of birth history), and incomplete data. Ultimately, the case group consisted of 233 singleton children with idiopathic congenital clubfoot.

The control group comprised 232 singleton children without congenital anomalies, born in 2003 or later, whose mothers resided within the study region. This region included all provinces of southern Vietnam and the Central Highlands, as most congenital and acquired musculoskeletal patients from this area received care at the Hospital for Traumatology and Orthopaedics in Ho Chi Minh City. Both the case group and the control group met the estimated sample size of 194 cases for each group.

The study was conducted in accordance with the Declaration of Helsinki and approved by the IRB of the Hospital for Traumatology and Orthopaedics with decision number 973/BVCTCH after the project of this study, which involved the patients had been officially presented. The informed consent has been obtained and signed by the parents of all patients to publish this paper.

Methods

This retrospective case-control study design was employed to compare the prevalence of child and maternal characteristics between children with idiopathic CCF and those without congenital anomalies. Questionnaires of birth history data were obtained directly from the mothers of the children. The control group was selected based on a standardized medical record template and included children who visited the hospital were hospitalized or were siblings of patients, provided they had no congenital anomalies, besides having the same ages and region as the case group.

Data processing and statistical analysis

Data were processed using Stata 10.0 (StatCorpLLC, College Station, TX). Descriptive statistics included the calculation of sex ratios and the proportion of variables associated with children and mothers. Analytical statistics were conducted to assess the associations between maternal and child variables and the occurrence of CCF. These associations were evaluated using odds ratios and Fisher's exact test; significance was considered at p values < 0.05 for all statistical tests.

## Results

Characteristics of CCF

The characteristics of CCF are described in Table 1. Among 233 CCF patients, 161 (69.1%) were male, twice as many as the 72 female patients (30.9%). Bilateral CCF occurred in 117 patients (50.2%), like unilateral cases in 116 patients (49.8%). Among unilateral cases, right-sided clubfoot (70 patients, 60.3%) predominated over left-sided clubfoot (46 patients, 39.7%).

**Table 1:** Phenotypic data of clubfoot (n = 233)

Phenotypic Data	Number of Patients
Sex	
Male	161 (69.1%)
Female	72 (30.9%)
Laterality	
Unilateral	
Left	46 (19.7%)
Right	70 (30.1%)
Bilateral	117 (50.2%)

Potential risk factors in children and mothers

Table 2 describes characteristics of children and mothers that may represent risk factors. Male children showed a significantly higher risk of CCF compared to female children, with an odds ratio (OR) of 1.75 and a 95% confidence interval (CI) of 1.17-2.61. Preterm birth (<37 weeks) increased the risk, but this was not statistically significant (OR = 1.88; 95% CI = 0.88-4.15). Similarly, low birth weight (≤2500 g) also increased the risk, though this was not statistically significant (OR = 1.62; 95% CI = 0.85-3.11). The breech presentation showed a significantly elevated risk of clubfoot, with p = 0.001.

**Table 2:** Characteristics of children and mothers (*), (**) Some characteristics among cases and controls were missing; (***) Fisher’s exact test was used due to the absence of breech presentation cases among controls. OR: Odd ratio; 95% CL: 95% confidence interval

Characteristics	Cases (n = 233) (*)	Controls (n' = 232) (**)	OR; 95% CI
Sex			
Male	161 (69.1%)	130 (56.0%)	1.75; 1.17–2.61
Female	72 (30.9%)	102 (44.0%)	
Gestational age (weeks)			
<37	23 (10.0%)	13 (5.6%)	1.88; 0.88–4.15
≥37	206 (90.0%)	219 (94.4%)	
Birth weight (grams)			
≤2500	30 (13.3%)	20 (8.7%)	1.62; 0.85–3.11
>2500	195 (86.7%)	211 (91.3%)	
Breech presentation			
Yes	10 (4.5%)	0 (0.0%)	p=0.001 (***)
No	213 (95.5%)	225 (100.0%)	
Maternal age at birth			
≤23	145 (62.5%)	146 (62.9%)	0.85; 0.55–1.30
24–34	65 (28.0%)	56 (24.2%)	Reference
≥35	22 (9.5%)	30 (12.9%)	0.63; 0.32–1.21
Birth order			
First	135 (58.7%)	121 (52.1%)	0.76; 0.52–1.12
Subsequent	95 (41.3%)	111 (47.8%)	
Delivery			
Vaginal	174 (75.6%)	170 (73.6%)	0.89; 0.57–1.39
Cesarean	56 (24.4%)	61 (26.4%)	
Birth season			
Dry (Nov–Apr)	106 (45.5%)	119 (53.4%)	1.37; 0.93–2.01
Rainy (May–Oct)	127 (54.5%)	104 (46.6%)	
Living place			
Ho Chi Minh City	49 (21.0%)	87 (37.5%)	Reference
Western area	89 (38.2%)	71 (30.6%)	2.22; 1.39–3.55
Eastern area	68 (29.2%)	61 (26.3%)	1.97; 1.21–3.23
Highland	27 (11.6%)	13 (5.6%)	3.68; 1.74–7.79

Additionally, subsequent births tended to decrease risk, but this was not statistically significant (OR = 0.76; 95% CI = 0.52-1.12). Mode of delivery (vaginal or cesarean) did not show significant effects (OR = 0.89; 95% CI = 0.57-1.39). Maternal age at delivery, whether young (≤23 years) or older (≥35 years), showed no significant differences compared to standard maternal age (24-34 years), although older maternal age tended to reduce risk (OR = 0.63; 95% CI = 0.32-1.21). Children born in the rainy season were more likely to have CCF than those born in the dry season, though this was not statistically significant (OR = 1.37; 95% CI = 0.93-2.01).

Compared to mothers residing in Ho Chi Minh City, the risk increased significantly for mothers living in the western region (OR = 2.22; 95% CI = 1.39-3.55), eastern region (OR = 1.97; 95% CI = 1.21-3.23), and Central Highlands (OR = 3.68; 95% CI = 1.74-7.79). When compared to the Central Highlands, the risk of CCF was reduced for mothers residing in the western region (OR = 0.60; 95% CI = 0.28-1.26) and the eastern region (OR = 0.53; 95% CI = 0.25-1.14), though these findings were not statistically significant.

## Discussion

There are no official publications reporting the prevalence of CCF in Vietnam; however, this figure is estimated to be approximately 0.1%, according to Evans et al. [3]. Among the 233 patients in this study, bilateral CCF accounted for 50.2%, while right-sided CCF was more common than left-sided, at 30.1% compared to 19.7%. These findings align with those of Kancherla et al. [5], who observed that approximately half of the cases were bilateral, with a trend of unilateral cases predominantly affecting the right side in Iowa from 1997 through 2005. Similarly, Parker et al. [6] reported a similar trend, with bilateral CCF comprising 54.1% of cases in a population-based study in the USA.

Table 2 demonstrates that male sex is a risk factor for CCF, with an OR of 1.75 (95% CI: 1.17-2.61). According to Kancherla et al. [5], both unadjusted and adjusted analyses indicated a significantly higher risk of idiopathic CCF in male children. Parker et al. [6] also identified a strong association between male sex and CCF (OR = 1.67; 95% CI: 1.58-1.76). Additionally, Carey et al. [4] identified male sex and Indigenous ethnicity as significant risk factors in their study of idiopathic CCF in Western Australia.

Preterm birth (<37 weeks) was associated with an increased risk of CCF, though not statistically significant (OR = 1.88; 95% CI: 0.88-4.15). Parker et al. [6] reported a strong association between preterm birth and idiopathic CCF. However, several studies did not consider preterm birth as a risk factor. For example, Kancherla et al. [5] limited their study to full-term cases to reduce methodological biases, while Carey et al. [4] suggested that prolonged gestation may contribute to intrauterine constraint, a potential risk factor.

Low birth weight (≤2500 g) was associated with a higher risk of CCF than normal birth weight (>2500 g), though this finding was not statistically significant (OR = 1.62; 95% CI: 0.85-3.11). Parker et al. [6] identified a strong association between low birth weight and idiopathic CCF, but this relationship has not been consistently reported in other studies [4,5,9]. Carey et al. [4] noted that higher birth weight could also contribute to intrauterine constraint.

A significant association was observed between breech presentation and CCF (p = 0.001). This finding supports Nguyen et al.'s study [14], which identified a significant increase in CCF risk associated with breech presentation (p = 0.033). However, due to the absence of breech presentation cases in the control group, the OR could not be calculated, highlighting the need for larger-scale, population-based studies. Parker et al. [6] also found a strong association between breech presentation and idiopathic CCF, with similar trends reported by Lochmiller et al. [15]. However, Kancherla et al. [5] observed a lower OR for breech presentation, likely due to limited case numbers. Carey et al. [4] suggested that intrauterine constraint, including breech presentation, may not significantly contribute to CCF risk after multivariate analysis.

Regarding maternal characteristics, Table 2 shows that advanced maternal age (≥35 years) and younger maternal age (≤23 years) were associated with reduced risks of CCF compared to the reference age group (24-34 years), though these associations were not statistically significant (OR = 0.63, 95% CI: 0.32-1.21, and OR = 0.85, 95% CI: 0.55-1.30, respectively). The high proportion of young mothers in both cases (62.5%) and control (62.9%) groups may reflect cultural and socioeconomic factors. Further research using a broader population and defining younger maternal age as <20 years is needed to better assess this relationship. Studies on maternal age and CCF have reported inconsistent findings. Parker et al. [6] observed a reduced risk with younger maternal age (OR = 0.90, 95% CI: 0.83-0.96) after adjustment, whereas univariate analysis suggested an increased risk (OR = 1.14, 95% CI: 1.08-1.20). Kancherla et al. [5] reported a significant reduction in risk for mothers aged ≥35 compared to those aged 20-34. Conversely, Nguyen et al. [14] identified a significant increase in risk for mothers under 25 years of age (p = 0.026).

Multiparity (having subsequent births) was associated with a reduced risk of CCF compared to primiparity (first births), though not statistically significant (OR = 0.76; 95% CI: 0.52-1.12). This trend has been confirmed in several studies [6,16]. Parker et al. [6] found a strong association between higher parity and reduced CCF risk, while Cardy et al. [16] reported a significant association between pregnancy frequency and idiopathic CCF in multivariate analysis. However, other studies have found no significant relationship between parity and CCF [4,5,12,13,17].

The mode of delivery, whether vaginal or cesarean, was not significantly associated with CCF (OR = 0.89; 95% CI: 0.57-1.39). Recent studies have shown limited interest in delivery mode as a risk factor, likely due to the increasing prevalence of elective cesarean sections. However, Cardy et al. [16] in the United Kingdom found a significant association between cesarean delivery and idiopathic CCF in multivariate analysis; the observed association may be due to the lower-than-expected frequency of caesarean births among controls.

In southern Vietnam and the Central Highlands, the study area has two distinct seasons: the dry season (November to April) and the rainy season (May to October). Table 2 indicates a higher incidence of CCF among children born during the rainy season compared to the dry season, though this was not statistically significant (OR = 1.37; 95% CI: 0.93-2.01). Most studies have found no association between birth season and idiopathic CCF [1,4,5,15].

Because Ho Chi Minh City is the biggest urban city in the study region, as well as in Vietnam, maternal residence was categorized geographically into Ho Chi Minh City, the western region, the eastern region, and the Central Highlands. Table 2 shows a significantly higher risk of CCF for mothers living in the western region (OR = 2.22; 95% CI: 1.39-3.55), eastern region (OR = 1.97; 95% CI: 1.21-3.23), and Central Highlands (OR = 3.68; 95% CI: 1.74-7.79) compared to those in Ho Chi Minh City. Compared to the Central Highlands, the risk decreased in the western region (OR = 0.60; 95% CI: 0.28-1.26) and eastern region (OR = 0.53; 95% CI: 0.25-1.14), though these findings were not statistically significant. These results suggest that maternal socioeconomic conditions, healthcare access, and education levels may influence CCF risk.

Other maternal characteristics, such as smoking, diabetes, and marital status, were not investigated in this study due to their low prevalence among young Vietnamese women. Similarly, paternal characteristics were not assessed, and paternal risk factors remain largely undefined. Furthermore, a limitation of this study was its reliance on birth history information provided directly by the mothers, rather than utilizing data from a formal birth registry, as has been employed in more recent studies [18-20].

## Conclusions

This study identified significant associations between congenital clubfoot and infant sex, breech presentation, and maternal geographical location. Further population-based studies are needed to explore these risk factors on a broader scale.
